# Prenatal and Postnatal Exposure to Phthalate Esters and Asthma: A 9-Year Follow-Up Study of a Taiwanese Birth Cohort

**DOI:** 10.1371/journal.pone.0123309

**Published:** 2015-04-13

**Authors:** Hsiu Ying Ku, Pen Hua Su, Hui Ju Wen, Hai Lun Sun, Chien Jen Wang, Hsiao Yen Chen, Jouni J. K. Jaakkola, Shu-Li Wang, TMICS Group

**Affiliations:** 1 Graduate Institute of Life Science, National Defense Medical Center, Taipei, Taiwan; 2 Division of Environmental Health & Occupational Medicine, National Health Research Institutes, Miaoli, Taiwan; 3 Division of Genetics, Department of Pediatrics, Chung Shan Medical University Hospital, Taichung, Taiwan; 4 Department of Pediatrics, Chung Shan Medical University Hospital, Taichung, Taiwan; 5 Center for Environmental and Respiratory Health Research, University of Oulu, Oulu, Finland; 6 Medical Research Center, Oulu, Finland; 7 Department of Public Health, College of Public Health, China Medical University, Taichung, Taiwan; 8 School of Public Health, National Defense Medical Center, Taipei, Taiwan; Taipei City Hospital, TAIWAN

## Abstract

Previous studies have shown that phthalate exposure in childhood is associated with the development of respiratory problems. However, few studies have assessed the relative impact of prenatal and postnatal exposure to phthalates on the development of asthma later in childhood. Therefore, we assessed the impact of prenatal and postnatal phthalate exposure on the development of asthma and wheezing using a Taiwanese birth cohort. A total of 430 pregnant women were recruited, and 171 (39.8%) of them had their children followed when they were aged 2, 5, and 8 years. The International Study of Asthma and Allergies in Childhood questionnaire was used to assess asthma and wheezing symptoms and serum total immunoglobulin E levels were measured at 8 years of age. Urine samples were obtained from 136 women during their third trimester of pregnancy, 99 children at 2 years of age, and 110 children at 5 years. Four common phthalate monoester metabolites in maternal and children’s urine were measured using liquid chromatography-electrospray ionization-tandem mass spectrometry. Maternal urinary mono-benzyl phthalate [MBzP] concentrations were associated with an increased occurrence of wheezing in boys at 8 years of age (odds ratio [OR] = 4.95 (95% CI 1.08–22.63)), for upper quintile compared to the others) after controlling for parental allergies and family members' smoking status. Urinary mono-2-ethylhexyl phthalate [MEHP] levels over the quintile at 2-year-old were associated with increased asthma occurrence (adjusted OR = 6.14 (1.17–32.13)) in boys. Similarly, the sum of di-2-ethyl-hexyl phthalate [DEHP] metabolites at 5 years was associated with asthma in boys (adjusted OR = 4.36 (1.01–18.86)). Urinary MEHP in maternal and 5-year-old children urine were significantly associated with increased IgE in allergic children at 8 years. Prenatal and postnatal exposure to phthalate was associated with the occurrence of asthma in children, particularly for boys.

## Introduction

Over the past few decades, the prevalence of childhood asthma has been increasing throughout the world [1]. However, the prevalence of asthma has increased faster in Taiwan than in most countries, possibly due to increased exposure to environmental factors [1]. It has been hypothesized that the increase in asthma is attributable to exposure to endocrine disruptors that act as adjuvants to immunoglobulin E (IgE)-dependent mechanisms and allergen-specific T helper type 2 (Th2) immune responses [2,3].

Phthalate, an endocrine-disrupting chemical, is a widely used plasticizer that is added to consumer products, acts to soften plastics, and may augment allergic processes [3]. The most commonly used phthalates are di-2-ethyl-hexyl phthalate (DEHP) and benzyl butyl phthalate (BBzP), which is the most commonly found phthalate in indoor settings. Polyvinyl chloride (PVC) flooring is known to be a source for the 2 phthalates in indoor dust [4]. Some phthalates, including diethyl phthalate (DEP), are used as solvents in personal care products and are associated with decreased lung function in men [5]. Although the primary route of exposure to phthalates is through contaminated food, children have a higher total phthalate intake than adults, likely as a result of mouthing plastics and ingesting indoor dust [6]. The intensive use of plastic materials may be related to the increased exposure to phthalate esters observed in Taiwan, thereby increasing the risk of adverse effects of phthalates, particularly in pregnant women and children [7,8].

Experimental studies have proposed that DEHP may act as an adjuvant to promote allergic asthma. When DEHP and allergen exposure are combined, DEHP exposure has been found to increase levels of immunological and inflammatory markers, including total IgE, interleukin-4, interferon (IFN)-γ levels, and eosinophil counts [9]. A recent study also found that DEHP and BBzP may promote the Th2 response to increase allergies through the suppression of IFN-α/IFN-β expression and stimulation of T-cell responses [10]. Epidemiological studies have consistently shown that early phthalate exposure is associated with childhood asthma [11,12, 13]. A Norwegian study has observed that the risk of asthma in children is increased in the highest quartiles of mono-carboxyoctyl phthalate and mono-carboxynonyl phthalate exposure [12]. Whyatt *et al*. reported that prenatal exposure to BBzP and di-n-butyl phthalate (DnBP) may increase the risk of asthma among children at 5 to 11 years of age [13].

A nested case-control study demonstrated a relationship between phthalates in dust at home and asthma occurrence in children [14]. However, the effects of phthalate exposure during fetal and early childhood on the development of asthma later in life remain unknown. The immune system matures at 2 years of age; infants are particularly susceptible to environmental exposures during the fast developmental stage [15]. Therefore, our aim was to assess the association between prenatal and postnatal exposure to phthalates with the risk of asthma and wheezing using a 9-year birth cohort study.

## Materials and Methods

### Ethics statement

The study protocol was approved by the Institutional Review Board of the National Health Research Institutes, Taiwan. Prior to participating in the study, the pregnant women and/or children’s caretakers provided written informed consent during three visits, having received detailed explanations of the benefits and risks.

### Study population and data collection

**Fig 1:**
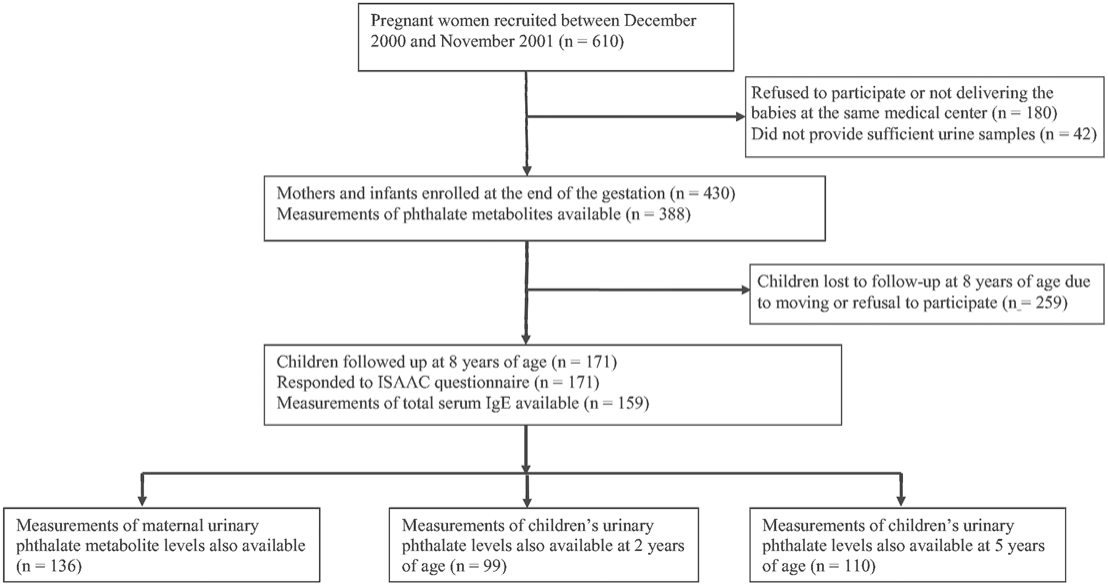
Flow chart of the recruitment process of pregnant women and follow up of their children. Pregnant women were invited to participate in the study. During the follow-up period, phthalate metabolites present in maternal and children’s urine were measured. At 8 years of age, the presence of asthma and wheezing was assessed using the International Study of Asthma and Allergies in Childhood (ISAAC) questionnaire.

This study was performed using data from an ongoing birth cohort study in central Taiwan and served as pilot for Taiwan Maternal and Infant Cohort Study. The study participants were pregnant women without clinical complications (eclampsia or preeclampsia), who delivered their babies in a local hospital between December 1, 2000 and November 30, 2001 (n = 610) [16,17]. We recruited 430 pregnant women and followed up their children at the ages of 2, 5, and 8 years in 2003, 2006, and 2009, respectively. We recruited the pregnant women during the third trimester of their pregnancies because pregnant women tended not to return for follow up when we attempted to recruit them during the first trimester. For example, they changed their childbirth hospital in the later stages of pregnancy to be closer to their parental homes. After receiving detailed explanations of the study and completing written informed consent forms, 430 women were invited to provide urine samples and information regarding their demographic and disease history (n = 388). A total of 171 (39.8%) children were evaluated at the age of 8 years to investigate for asthma symptoms; 259 children were lost to follow up because of moving or their parents’ or caregivers’ refusal to participate. At the follow-up visit, primary caretakers completed a questionnaire, and blood and urine samples were collected from the children. After excluding children that were lost to follow up, 136 maternal urine samples obtained during the third trimester of pregnancy, 99 urine samples obtained from children at 2 years of age, and 110 urine samples obtained from the children at 5 years of age were analyzed. A flow chart depicting the details of the study population is shown in Fig 1.

### Disease definition and symptoms

The International Study of Asthma and Allergies in Childhood (ISAAC) is a standardized and validated method to investigate variations in childhood allergies [18]. We used the protocol of the ISAAC Chinese version to assess asthma in children at 8 years of age. A child was considered to have asthma if the primary caretakers responded positively to the question “Has your child ever been diagnosed with asthma by a physician?” and was considered to have a history of wheezing if the primary caretaker responded positively to the question “Has your child ever had a chest wheeze when he/she did not have a flu?” Children were classified as having a parental history of asthma if at least one parent responded positively to the question “Have you ever been diagnosed with asthma by a physician?”

### Analysis of total serum immunoglobulin E levels

To determine serum IgE levels in 8-year-old children, blood samples (0.5 mL) obtained via venipuncture were centrifuged and the sera stored at −20°C prior to analysis. Serum total IgE (tIgE) levels were measured using the ADVIA Centaur chemiluminescence immunoassay system (Siemens Healthcare Diagnostics; Deerfield, IL, USA). The assay range was approximately 1.5–3000 IU/mL.

### Analysis of phthalate metabolites

Four phthalate monoester metabolites (mono-2-ethylhexyl phthalate [MEHP], mono-benzyl phthalate [MBzP], mono-butyl phthalate [MBP], and mono-ethyl phthalate [MEP]) that are representative of exposure to four commonly used phthalates (DEHP, BBzP, di-butyl phthalate [DBP], and DEP) were measured in maternal and children’s urine samples (S1 Table). Details of the analysis and quality control procedures for the pregnant women’s samples have been described previously [19]. Analysis of single urine samples from children aged 2, 5, and 8 years was performed according to a modified method previously described by Koch *et al*. [20]. In brief, 0.1 mL urine sample aliquots containing 1 M ammonium acetate (20 μL), β-glucuronidase (10 μL), and a mixture of isotopic phthalate metabolite standards were prepared. The samples were incubated at 37°C for 1.5 hours. Subsequent to hydrolysis, each sample was injected with 270 μL solvent (5% acetonitrile and 0.1% formic acid) in glass screw-cap vials and mixed for quantitative liquid chromatography-electrospray ionization-tandem mass spectrometry. Urinary creatinine levels were measured at the Kaohsiung Medical University Chung-Ho Memorial Hospital using a spectrophotometric method. Creatinine-corrected adjustment for each individual was used to examine associations between the compound exposure and asthma symptoms. If we used indirect adjustment by including urinary creatinine concentrations and uncorrected phthalate levels in multiple regression analyses, similar associations between the phthalate level and asthma status were found.

### Statistical analysis

The differences between pregnant women lost to follow up and those included in the analysis were estimated using independent t-tests for continued variables and Chi-square tests for categorical variables. In our preliminary analyses, we applied exposure categories based on tertiles, quartile, and quintiles of biomarker concentrations. We found that increased risk was first observed when exposure levels achieved the highest quintile. We believe that this may have resulted from lower levels of MBzP and MEP in our subjects relative to those of other studies [21,22]. Therefore, we divided the participants into 2 groups: the ‘highest quintile” group, which consisted of the participants within the 80^th^ to 100^th^ percentiles, and the reference group, which consisted of the participants that were below the 80^th^ percentile. The adjusted odds ratio (aOR) for asthma or wheezing was calculated using logistic regression models adjusted for parental allergies and family members’ smoking status. We defined parental allergies as: at least one parent having been diagnosed with any of asthma, dermatitis, rhinitis, or conjunctivitis. Multiple linear regression analyses were performed to examine the association between different phthalate metabolites and total IgE levels, after controlling for sex and parental allergies. Measured phthalate metabolite and total IgE levels were transformed by taking the base 10 log to fit normal distributions. The creatinine-corrected adjusted urinary concentrations of MEHP, mono-2-ethyl-5-oxohexyl phthalate, and mono-2-ethyl-5-hydroxyhexyl phthalate were combined to assess DEHP exposure (ΣDEHP). Statistical analyses were carried out using SPSS 17.0 software (Chicago, IL, USA). Hsiu-Ying Ku, Hui-Ju Wen, and Shu-Li Wang had full access to all data in this study and took responsibility for the integrity of the data and accuracy of the data analysis.

## Results

### Characteristics of the study population

**Fig 2:**
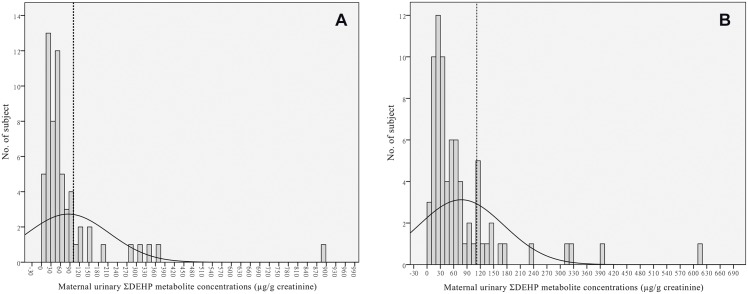
Distribution of urinary phthalate metabolite concentrations (μg/g creatinine) in pregnant women. The sum of di-2-ethylhexyl phthalate (ΣDEHP) metabolite concentrations was skewed to the right. Subjects in the highest quintile (80^th^ to 100th percentile) showed the strongest determinant of the outcomes of interest. (A). The cut-point for the highest quintile of maternal ΣDEHP metabolite concentration was 101 μg ΣDEHP/g creatinine in boys. (B). The cutoff was 112 μg ΣDEHP/g creatinine in girls.

Demographic characteristics of pregnant women with children who were followed up successfully at 8 years (n = 171) and those lost to follow up (n = 217) are shown in Table 1. Pregnant women who were followed up tended to be educated to a higher level (i.e., ≥university) relative to those lost to follow up. The distribution of phthalate metabolite levels was skewed to the right (Fig 2). We found that pregnant women who were lost to follow up tended to exhibit higher geometric mean concentrations on four phthalate metabolites, with the exception of MEP concentration.

**Table 1:** Demographics and maternal urinary phthalate metabolite concentrations (μg/g creatinine) in pregnant women with and without children who were followed up for asthma at 8 years of age.

Variables	Pregnant women with children lost to follow up (n = 217)	Pregnant women with children followed up (n = 171)
Mean age at delivery (y)	28.44 ± 4.29	29.10 ± 4.00
Age at delivery (y)		
≤35	205 (94.5%)	160 (93.6%)
>35	12 (5.5%)	11 (6.4%)
Maternal education		
≤High school	112 (51.6%)	70 (40.9%)
Junior college	78 (35.9%)	75 (43.9%)
≥University	27 (12.4%)	26 (15.2%)
Paternal education		
≤High school	105 (48.4%)	67 (39.2%)
Junior college	83 (38.2%)	76 (44.4%)
≥University	29 (13.4%)	28 (16.4%)
Family income per year (US Dollars)		
≤$20,000	96 (44.2%)	67 (39.2%)
$20,000–50,000	87 (40.1%)	76 (44.4%)
>$50,000	34 (15.7%)	28 (16.4%)
Smoking during pregnancy		
Yes	5 (3.5%)	0 (0%)
No	212 (96.5%)	171 (100%)
Passive smoking prior to pregnancy		
Yes	93 (42.9%)	81 (47.4%)
No	124 (57.1%)	90 (52.6%)
MEHP^a^ ^b^ (μg/g creatinine)	20.11 (17.83–22.70)	16.90 (14.49–19.72)
ΣDEHP^a^ ^b^ (μg/g creatinine)	54.24 (48.28–60.93)	50.22(42.22–59.72)
MBzP^a^ ^b^ (μg/g creatinine)*	18.76 (17.08–20.61)	15.48 (13.59–17.63)
MBP^a^ ^b^ (μg/g creatinine)	74.86 (66.73–83.97)	66.14 (56.06–78.03)
MEP^a^ ^b^ (μg/g creatinine)	61.37 (52.62–71.57)	65.15 (58.52–72.53)

Data are presented as number (%) or mean.

ΣDEHP, sum of metabolites of di-2-ethylhexyl phthalate; MEHP, mono-2-ethylhexyl phthalate; MBzP, mono-benzyl phthalate; MBP, mono-butyl-phthalate; MEP, mono-ethyl phthalate.

^a^Phthalate metabolite data available for n = 252 pregnant women with children lost to follow up and n = 136 pregnant women with followed-up children.

^b^Data are presented as geometric mean (95% confidence interval).

*Indicates a significant (*p* <0.05) difference between lost to follow up and followed up participants by Mann-Whitney U test.

### Childhood asthma risk

At 8 years of age, 32 of the 171 participating children (19%) showed a history of wheezing (24% of boys, 14% of girls; S2 Table). Boys were more likely to have allergic asthma relative to girls (19% and 10%, respectively). In order to explore potential confounding for associations between phthalate exposure and asthma, we examined differences in the distributions of possible confounders among children with and without asthma using Chi-square or Fisher’s exact tests (Table 2). There was a borderline significant difference in family members’ smoking status between asthmatic and non-asthmatic groups. In addition, based on current knowledge, gender and parental history of asthma are important predictors for asthma in children. Therefore, model covariates included parental allergies and family members’ smoking status for sex-stratified analyses. MEHP levels appeared to be higher in our children as compared to Danish ones (S3 Table).

**Table 2:** Characteristics of children at 8 years of age classified by asthma status (n = 171).

Variables	Children without asthma n (%)	Children with asthma n (%)	*p*-value^a^
Gender			0.09
Girls	82 (55.8%)	9 (37.5%)	
Boys	65 (44.2%)	15 (62.5%)	
Maternal education			0.56
≤High school	51 (35.2%)	10 (41.7%)	
Junior college	55 (37.9%)	10 (41.7%)	
≥University	39 (26.9%)	4 (16.7%)	
Maternal allergies			0.30
No	90 (61.2%)	12 (50.0%)	
Yes	57 (38.8%)	12 (50.0%)	
Paternal allergies			0.79
No	119 (81.0%)	19 (79.2%)	
Yes	28 (19.0%)	5 (20.8%)	
Parental allergies			0.46
No	73 (49.7%)	10 (41.7%)	
Yes	74 (50.3%)	14 (58.3%)	
Family income per year (US Dollars)			0.62
<US$20,000	49 (36.0%)	6 (31.6%)	
$20,000–50,000	49 (36.0%)	9 (47.4%)	
>$50,000	38 (27.9%)	4 (21.1%)	
Family members' moking status			0.06
No	79 (53.7%)	8 (33.3%)	
Yes	68 (46.3%)	16 (66.7%)	
Breastfeeding			0.67
≤3 months	116 (78.9%)	18 (75.0%)	
>3 months	31 (21.1%)	6 (25.0%)	
Water leakage at home (days/year)			0.49
0	130 (89.0%)	20 (83.3%)	
≥1	16 (11.0%)	4 (16.7%)	
Cockroaches observed at home (cockroaches/month)			0.96
0–2	57 (39.6%)	9 (39.1%)	
≥3	87 (60.4%)	14 (60.9%)	
Number of walls with mold at home			0.46
0	66 (45.2%)	10 (41.7%)	
≥1	80 (54.8%)	14 (58.3%)	
Serum total IgE levels (IU/mL)	74.8 ± 22.74	132.5 ± 90.58	0.27^b^

Data are presented as number (%) or median ± standard error.

IgE, immunoglobulin E.

^a^ *p* values for differences between groups were estimated using Chi-square (Fisher’s exact) test.

^b^ *p* values for the difference between non-asthmatic children and asthmatic children using a Mann-Whitney U test.

### Phthalates and asthma

The association between different phthalate metabolites and asthma or wheezing in children is shown in Table 3. An overview of the results indicated positive associations between maternal phthalate metabolite concentrations and ever wheezing in children of both sexes, with the exception of MBP. In boys, there was a significant association between the prevalence of ever wheezing and a maternal MBzP concentration greater than the 80th percentile (aOR = 4.95; 95% CI: 1.08–22.63), compared with the reference group with MBzP levels less than or equal to the 80th percentile, after adjusting for maternal and paternal allergies and family members’ smoking status. In addition, there was a positive association of borderline significance between the prevalence of ever wheezing and maternal ΣDEHP metabolite concentrations in boys (aOR = 4.12; 95% CI: 0.87–19.61). In boys, the upper quintile of 2-year-old MEHP concentrations had a significant odds ratio for asthma at 8 years (aOR = 6.14; 95% CI: 1.17–32.13) and similar association patterns were found in 5-year-old MEHP. Boys in the upper quintile concentration of 5-year-old ΣDEHP were at an approximately four fold increased risk of asthma at 8 years of age (aOR = 4.36; 95% CI: 1.01–18.86), relative to the reference group. Similarly, we observed significantly increased odds ratios for asthma in the upper quintile of 5-year-old MEP concentration in boys (aOR = 8.92; 95% CI: 1.87–42.54). There was also a borderline significant positive association between the prevalence of ever wheezing and 5-year-old MEP (aOR = 4.22; 95% CI: 0.87–20.51). In girls, no significant association was observed between phthalate metabolite concentrations and risk of asthma or wheezing.

**Table 3:** Adjusted odds ratios for wheezing and asthma occurrence at 8 years for the upper quintile in urinary phthalate metabolite concentrations *in utero* and at 2 and 5 years of age.

	Phthalate metabolite upper quintile	Boys				Upper quintile	Girls				Upper quintile	Total			
		Wheezing		Asthma			Wheezing		Asthma			Wheezing		Asthma	
		aOR	95% CI	aOR	95% CI		aOR	95% CI	aOR	95% CI		aOR	95% CI	aOR	95% CI
**A. Urinary metabolites during the 3** ^**rd**^ **trimester**															
	n = 62					n = 74					n = 136				
MEHP	>33.5	2.38	(0.48–11.84)	0.93	(0.16–5.19)	>38.5	1.09	(0.11–10.80)	1.04	(0.10–10.43)	>42.0	1.68	(0.47–6.02)	0.96	(0.24–3.81)
ΣDEHP	>101.3	**4.12** ^#^	**(0.87–19.61)**	0.77	(0.14–4.28)	>112.7	2.80	(0.42–18.69)	0.74	(0.07–7.39)	>108.4	**3.12** ^#^	**(0.98–9.98)**	0.81	(0.21–3.14)
MBzP	>23.9	**4.95** *	**(1.08–22.63)**	0.83	(0.15–4.66)	>31.9	1.00	(0.10–9.98)	1.00	(0.10–10.08)	>32.3	1.71	(0.46–6.38)	0.68	(0.14–3.38)
MEP	>118.6	1.17	(0.20–6.80)	0.88	(0.16–4.80)	>121.2	2.94	(0.44–19.70)	-	-	>119.3	1.54	(0.44–5.40)	0.48	(0.10–2.30)
MBP	>142.0	0.35	(0.04–3.17)	-	-	>180.8	0.99	(0.10–9.68)	0.72	(0.07–7.12)	>158.3	0.66	(0.14–3.22)	0.23	(0.03–1.83)
**B. Urinary metabolites at 2 years**															
	n = 50					n = 49					n = 99				
MEHP	>31.8	2.39	(0.47–12.23)	**6.14** *	**(1.17–32.13)**	>43.2	1.05	(0.10–11.41)	1.46	(0.10–21.47)	>31.9	1.74	(0.46–6.58)	2.94	(0.70–12.26)
ΣDEHP	>322.9	1.03	(0.17–6.23)	3.78	(0.64–22.30)	>323.7	0.99	(0.09–10.33)	-	-	>316.8	1.00	(0.24–4.13)	1.68	(0.38–7.48)
MBzP	>27.3	1.47	(0.24–9.10)	1.48	(0.24–9.34)	>19.9	-	-	-	-	>19.2	0.57	(0.11–2.96)	0.71	(0.13–3.77)
MEP	>54.3	-	-	-	-	>83.0	-	-	-	-	>78.9	0.27	(0.03–2.32)	-	-
MBP	>283.4	0.40	(0.04–3.75)	1.33	(0.22–8.06)	>276.6	1.13	(0.11–11.70)	-	-	>277.4	0.59	(0.12–2.93)	0.75	(0.14–3.96)
**C. Urinary metabolites at 5 years**															
	n = 55					n = 55					n = 110				
MEHP	>25.2	0.86	(0.15–4.88)	2.61	(0.60–11.37)	>25.1	-	-	-	-	>24.6	0.59	(0.12–2.91)	1.27	(0.36–4.49)
ΣDEHP	>341.8	1.70	(0.35–8.24)	**4.36** *	**(1.01**–**18.86)**	>284.7	1.40	(0.12–15.98)	0.96	(0.09–9.98)	>290	1.59	(0.43–5.86)	**3.48** *	**(1.10**–**10.99)**
MBzP	>37.8	-	-	0.91	(0.16–5.25)	>29.2	-	-	-	-	>31.2	-	-	0.43	(0.09–2.14)
MEP	>56.9	**4.22** ^#^	**(0.87**–**20.51)**	**8.92** *	**(1.87**–**42.54)**	>51.5	1.74	(0.14–21.20)	-	-	>40.3	2.85	(0.81–10.04)	**2.74** ^#^	**(0.85**–**8.90)**
MBP	>201.5	0.41	(0.05–3.74)	1.50	(0.32–7.04)	>140.5	-	-	0.76	(0.07–8.55)	>209	0.23	(0.03–1.90)	0.66	(0.17–2.64)

Data are adjusted for parental allergies and family members' smoking status.

Urinary metabolites are measured as μg metabolite/g creatinine.

^a^OR: adjusted odds ratio; CI: confidence interval; MEHP: mono-2-ethylhexyl phthalate; ΣDEHP: sum of metabolites of di-2-ethylhexyl phthalate; MBzP: mono-benzyl phthalate; MEP: mono-ethyl phthalate; MBP: mono-butyl-phthalate.

*Indicates a significant (*p* <0.05) finding;

^#^indicates a borderline significant (*p* <0.10) finding.

### Phthalate metabolites and total immunoglobulin E levels

Log_10_-transformed concentrations of 8-year-old serum total IgE and creatinine-corrected urine phthalate metabolite were applied for further analysis. The unstandardized regression coefficients (B) for tIgE at 8 years of age, according to urine phthalate metabolite concentrations in pregnant women and children aged 2 and 5 years are shown in Table 4. A significant positive association was observed between maternal MBzP concentrations and tIgE (per log-unit: B = 0.39; *p* = 0.03) in 8-year-old children after adjusting for gender and paternal allergies. In allergic children, higher MEHP concentrations in pregnant women and 5-year-old children were positively correlated with tIgE levels at 8 years of age (per log-unit: B = 0.50, *p* <0.05 and B = 0.36, *p* <0.05, respectively). A significant positive correlation between MBzP levels at 2 years of age and tIgE in allergic children (per log-unit: B = 0.04, *p* = 0.02) was also observed. However, no association was observed in non-allergic children.

**Table 4:** Association between maternal and children’s log-transformed urinary phthalate metabolite concentrations (μg/g creatinine) and log-transformed total serum immunoglobulin E levels (IU/ml) in 8-year-old children.

Phthalate metabolite	Allergic children^a^	Non-allergic children	All children
**A. Urinary metabolites during the 3** ^**rd**^ **trimester**			
	n = 72	n = 58	n = 130
MEHP	**0.50 (0.02)** *	0.24 (0.26)	0.38 (0.16)
ΣDEHP	0.20 (0.32)	0.12 (0.51)	0.03 (0.89)
MBzP	0.20 (0.94)	0.22 (0.39)	**0.39 (0.03)** *
MEP	0.11 (0.55)	0.43 (0.09)	0.10 (0.58)
MBP	0.20 (0.34)	0.23 (0.20)	0.06 (0.72)
**B. Urinary metabolites at 2 years**			
	n = 56	n = 36	n = 92
MEHP	0.07 (0.77)	0.36 (0.18)	0.08 (0.66)
ΣDEHP	0.28 (0.36)	0.23 (0.36)	0.01 (097)
MBzP	**0.40 (0.02)** *	0.16 (0.32)	0.20 (0.11)
MEP	−0.29 (0.15)	−0.20 (0.29)	−0.23 (0.10)
MBP	−0.27 (0.37)	0.21 (0.41)	0.04 (0.85)
**C. Urinary metabolites at 5 years**			
	n = 63	n = 40	n = 103
MEHP	**0.36 (0.04)** *	−0.22 (0.20)	0.10 (0.43)
ΣDEHP	0.29 (0.16)	−0.14 (0.53)	0.14 (0.35)
MBzP	−0.03 (0.90)	0.09 (0.65)	0.03 (0.85)
MEP	−0.14 (0.43)	0.18 (0.30)	0.01 (0.97)
MBP	0.21 (0.29)	0.18 (0.51)	0.22 (0.17)

Data are presented as unstandardized regression coefficient (B) and *p*-value.

Associations were adjusted for gender and parental allergies.

MEHP: mono-2-ethylhexyl phthalate; ΣDEHP: sum of metabolites of di-2-ethylhexyl phthalate; MBzP: mono-benzyl phthalate; MEP: mono-ethyl phthalate; MBP: mono-butyl-phthalate.

^a^Allergic children included children with asthma, dermatitis, rhinitis, and conjunctivitis.

*Indicates a significant (*p* <0.05) association, estimated using linear regression, between log-transformed phthalate metabolite concentrations and log-transformed serum immunoglobulin E concentrations.

## Discussion

To our knowledge, this is the first study to assess the effects of both prenatal and postnatal exposure to phthalates on the risk for childhood asthma. In this study, higher maternal MBzP levels were associated with an approximately five-fold increase in the odds of wheezing in boys and substantial increases in tIgE levels in 8-year-old children of both sexes. In addition, we found that increased maternal ΣDEHP metabolite concentrations were associated with increased risk of wheezing in boys, although the association only had borderline statistical significance. Maternal MEHP levels were positively associated with increased tIgE levels in allergic children. This indicates that IgE tends to increase with increasing DEHP and BBzP metabolite concentrations in these allergic children.

Wheezing is one of the earliest symptoms of asthma, and in young children, it results from narrowing of the airways of the lungs as a result of inflammation [23]. Children who experience persistent wheezing tend to have higher asthma rates later in life [24]. Jaakkola *et al*. reported that exposure to plastic wall material was associated with a three fold increase in risk of persistent wheezing in children [25]. A recent study found that maternal urinary MBzP concentration was associated with history of asthma-like symptoms and current asthma [13]. Similarly, Kolarik *et al*. showed that DEHP in indoor dust was correlated with wheezing in children in Bulgaria [26]. Furthermore, Just *et al*. reported a positive association between current MBzP concentrations and airway inflammation, particularly in children who wheezed [27].

Boys in the upper quintile of DEHP metabolite and MEP concentrations at 2 and 5 years of age were more likely to experience asthma during the follow-up period. These results are consistent with those of a previous case-control study of children in which risk of asthma was significantly associated with DEHP concentrations [14]. In addition, current DEP exposure was positively associated with airway inflammation in children and decreased forced expiratory volume in 1 second in adults [5,27]. However, DEP and BBzP air samples were shown to be significantly correlated with urinary metabolite concentrations, suggesting that inhalation of phthalates may be an important pathway to the development of allergic asthma [28].

Our data showed positive correlations between urinary DEHP metabolites at different ages and tIgE levels in allergic children at 8 years of age. In addition, urinary MBzP concentrations at 2 years of age were positively associated with tIgE levels in 8-year-old allergic children. A strong positive association has previously been reported to exist between airway reactivity and DEHP levels in combination with allergen exposure and tIgE levels [9]. In addition, an association between MBzP and tIgE levels in 2-year-old boys has been observed in Taiwan [29]. Allergic asthma is thought to be a chronic inflammatory disease that is mainly driven by an allergen-specific Th2 immune response [30]. Therefore, it has been hypothesized that certain phthalates may be a trigger for asthma-related airway inflammation and an increase in Th2 immunoglobulins including IgE [30]. Our findings show that DEHP and MBzP metabolites might trigger increased tIgE levels in children with allergic diseases. Therefore, it appears that the allergy status of children makes them vulnerable to the effects of phthalate exposure.

There is limited knowledge regarding the mechanism through which phthalate exposure *in utero* promotes allergies. DEHP can cross the placenta and potentially result in abnormal fetal development [31]. We have previously shown a statistically significant association between maternal DEHP metabolite concentrations and cord blood concentrations of sex hormones [19]. In addition, prenatal BBzP exposure may predispose children to allergic sensitization, perhaps through the disruption of maternal estrogen during pregnancy [32]. Sex hormones during pregnancy influence the risk of allergic diseases in children [33]. Therefore, *in utero* phthalate exposure could disrupt maternal sex hormone concentrations and affect the development of the immune system in children. Moreover, *in utero* exposure to BBzP may modify the expression of genes related to immunity, including cluster of differentiation (CD) 24, CD5, cathepsin E, protein tyrosine phosphatase receptor type C, and secretory leukocyte protease inhibitor, through epigenetic effects [34]. Besides, MEHP may trigger release of TNF-α and initiate a possible differentiation towards anti-inflammatory macrophages [35].

We report the results of a long-term cohort study in which maternal exposure levels were determined prior to the diagnosis of the children’s allergic disease. In addition, we studied the role of tIgE levels as a marker of allergy and assessed its association with different phthalate metabolites. A limitation of this study was the relatively small sample size, which may have resulted in insufficient statistical power to conduct further stratification analyses. However, despite the sample size, significant associations between phthalate metabolites and asthma incidence in children were observed. A second limitation of the study was that maternal urine samples were collected during the third trimester of pregnancy. It has been reported that the relatively high temporal variability in DEHP metabolites in pregnant women result in non-differential exposure misclassification of DEHP; therefore, bias has a minimal effect [36]. A third limitation was the high percentage (60%) of participants lost to follow up at 8 years of age. However, a higher proportion of pregnant women who returned for follow up (15%) were educated to higher levels relative to those lost to follow up (12%). Our results also showed that geometric mean values of phthalate metabolites (except for MEP) were usually lower in the follow-up group. In addition, we used lifetime prevalence of physician-diagnosed asthma in the ISAAC questionnaire for respondents but did not record the onset of asthma that is generally diagnosed after 5 years of age. However, non-random misclassification of the exposure was unlikely, as asthmatic and non-asthmatic people are equally likely to be misclassified according to exposure. Together with previous findings that pregnant women’s exposure to phthalates has been reported to associate with asthma symptoms in their children. Therefore, it is not likely that our conclusion was biased. Future study is directed to record time of the disease onset in consecutive follow-ups.

Concerning the prevalence of asthma and wheezing, asthma rate (14%) in the present study was similar to that found in an earlier similar survey conducted in the same area (15%) [37]. However, the prevalence of asthma was higher than the prevalence reported in nationwide study on school-age children in Taiwan (8.8%) [38]. The reason for this discrepancy likely lies in the fact that the participants in our study were living in urban areas where prevalence of asthma tended to be higher than in rural areas. As compared to the international data, the prevalence of wheezing in our study (19%) was similar to that reported in international data (16–29%) [39] However, asthma prevalence among boys in our study (19%) appeared to be higher than that reported in international data (8–12%) [1]. It is relatively difficult to distinguished between cold and wheezing symptoms for parents and primary caretakers in Taiwan with high incidence of flu and cold. Thus, the prevalence of wheezing could have been underestimated in our study.

## Conclusions

We provide new evidence that early exposure to certain phthalates may associate with allergic sensitization and play a role in the development of asthma during the first 8 years of life. This observation raises substantial public health concerns given the widespread use of phthalate esters and increasing prevalence of asthma in children.

## Supporting Information

S1 Table Pearson’s correlation between log-transformed phthalate metabolite concentrations in urine samples of mothers and children aged 2, 5, and 8 years. (DOC)

S2 Table Demographic characteristics and the prevalence of allergic diseases in 8-year-old children. (DOC)

S3 Table Pregnant women and children's geometric means of phthalate metabolites in different studies. (DOC)
