# Supplementary material for: Prenatal and Postnatal Exposure to Phthalate Esters and Asthma: A 9-Year Follow-Up Study of a Taiwanese Birth Cohort
Source: PLoS One. 2015 Apr 13;10(4):e0123309. doi: 10.1371/journal.pone.0123309 (PMC4395154; doi:10.1371/journal.pone.0123309)
Supplement: S1 Table — (DOC) [file pone.0123309.s001.doc]

**S1 Table.** Pearson’s correlation between log-transformed phthalate metabolite concentrations in urine samples of mothers and children aged 2, 5, and 8 years^a^

| Phthalate metabolite | Avg. % > LOD | Maternal vs. 2 y (n = 79) | Maternal vs. 5 y (n = 96) | Maternal vs. 8 y (n = 136) | 2 y vs. 5 y  (n = 92) | 2 y vs. 8 y  (n = 99) | 5 y vs. 8 y  (n = 110) |
| --- | --- | --- | --- | --- | --- | --- | --- |
| MEHP | 100 | 0.08 | 0.13 | −0.02 | −0.03 | 0.11 | 0.20* |
| ΣDEHP | 100 | 0.12 | 0.09 | 0.05 | −0.12 | 0.10 | 0.20* |
| MBzP | 100 | −0.15 | −0.11 | −0.03 | 0.14 | 0.08 | −0.09 |
| MEP | 100 | 1.00 | 0.14 | −0.03 | 0.10 | 0.11 | 0.07 |
| MBP | 100 | 0.09 | −0.09 | 0.18* | 0.32* | −0.11 | −0.10 |

Avg., average; ΣDEHP, sum of metabolites of di-2-ethylhexyl phthalate; LOD, limit of detection; MBP, mono-butyl phthalate; MBzP, mono-benzyl phthalate; MEHP, mono-2-ethylhexyl phthalate; MEP, mono-2-ethylhexyl phthalate

^a^ Pearson’s correlation coefficient (r)

* Indicates a statistically significant finding (*p* < 0.05)
