# Supplementary material for: Prenatal and Postnatal Exposure to Phthalate Esters and Asthma: A 9-Year Follow-Up Study of a Taiwanese Birth Cohort
Source: PLoS One. 2015 Apr 13;10(4):e0123309. doi: 10.1371/journal.pone.0123309 (PMC4395154; doi:10.1371/journal.pone.0123309)
Supplement: S2 Table — (DOC) [file pone.0123309.s002.doc]

**S2 Table.** Demographic characteristics and the prevalence of allergic diseases in 8-year-old children by sex

| Variables | Boys (n = 80) | | Girls (n = 91) | Total | *P* value^a^ |
| --- | --- | --- | --- | --- | --- |
| Maternal age at delivery (y) | 29.22 ± 3.42 | | 28.63 ± 3.84 | 28.89 ± 3.79 | 0.31 |
| Maternal age at delivery (y) |  | |  |  | 0.08^#^ |
| ≤35 | 68 (91.9%) | | 71 (82.6%) | 139 (86.9) |  |
| >35 | 6 (8.1%) | | 15 (17.4%) | 21 (13.1) |  |
| Maternal education |  | |  |  | 0.25 |
| ≤High school | 23 (29.5%) | | 38 (41.8%) | 61 (36.1%) |  |
| Junior college | 33 (42.3%) | | 32 (35.2%) | 65 (38.5%) |  |
| ≥University | 22 (28.2%) | | 21 (23.1%) | 43 (25.4%) |  |
| Children age (y) | 8.05 ± 0.42 | | 8.20 ± 0.26 | 8.13 ± 0.35 | **0.04*** |
| Parity (No.) |  | |  |  | 0.30 |
| 1 | 45 (57.0%) | | 43 (48.9%) | 88 (52.7%) |  |
| ≥2 | 34 (43.0%) | | 45 (51.1%) | 79 (47.3%) |  |
| Smoking status of family members | | |  |  | 0.41 |
| No | 38 (47.5%) | | 49 (53.8%) | 87 (50.9%) |  |
| Yes | 42 (52.5%) | | 42 (46.2%) | 84 (49.1%) |  |
| Total serum IgE levels in children (IU/ml)^b^ | 94.70 ± 45.10 | | 63.30 ± 20.50 | 78.10 ± 24.00 | 0.13 |
| Total serum IgE levels in children (IU/ml) | | | |  | 0.50 |
| ≤150 | | 47 (63.5%) | 59 (69.4%) | 90 (56.6%) |  |
| >150 | | 27 (36.5%) | 26 (30.6%) | 69 (43.4%) |  |
| Ever wheezing | | 19 (23.7%) | 13 (14.3%) | 32 (18.7%) | 0.10 |
| Allergic asthma | | 15 (18.8%) | 9 (9.9%) | 24 (14.0%) | <0.10^#^ |

Data are numbers (%) or means ± standard deviations

^a^ *P*-values for the difference between boys and girls aged 8 years using the t-test for continuous variables and the Chi-square or Fisher’s exact test for categorical variables

^b^ Medians ± standard errors

* Indicates statistical significance (*p* < 0.05) and ^#^ borderline significance (*p* < 0.10)
