# Supplementary material for: Prenatal and Postnatal Exposure to Phthalate Esters and Asthma: A 9-Year Follow-Up Study of a Taiwanese Birth Cohort
Source: PLoS One. 2015 Apr 13;10(4):e0123309. doi: 10.1371/journal.pone.0123309 (PMC4395154; doi:10.1371/journal.pone.0123309)
Supplement: S3 Table — (DOC) [file pone.0123309.s003.doc]

**S3 Table.** Pregnant women and children's geometric means of phthalate metabolites in different studies.

|  | Danish children  (2006–2007)^c^ | Present study  (2006–2007) | NHANES  (2003–2004)^d^ | Present study  (2000–2001) |
| --- | --- | --- | --- | --- |
| Phthalate | 4–9 y (n = 845)^a^ | 5–6 y (n = 110)^a^ | Pregnant women (n = 91)^b^ | Pregnant women (n = 388)^b^ |
| MEHP | 6.8 | 11.9 | --- | 18.40 |
| MBzP | 25 | 15.0 | 15.12 | 17.44 |
| MEP | 34 | 20.0 | 226.53 | 63.40 |
| MBP | 199 | 114.5 | 22.30 | 73.02 |

MBP, mono-butyl phthalate; MBzP, mono-benzyl phthalate; MEHP, mono-2-ethylhexyl phthalate; MEP, mono-2-ethylhexyl phthalate; NHANES, National Health and Nutrition Examination Survey

^a^ Concentration of phthalate metabolites in urine (µg/g creatinine)

^b^ Concentration of phthalate metabolites in urine (ng/ml)

^c^ Danish children (2006–2007) [21]

^d^ NHANES (2003–2004) [22]
